# A Case of Paraplegia, Occasioned by the Forcible Depression of Six Dorsal Vertebræ, All of Which Were Restored to Their Natural Situations in the Spinal Column, by the Process Employed for That Purpose

**Published:** 1823-03

**Authors:** Edward Harrison


ART. II.—*A Case of Paraplegia, occasioned by the forcible Depression of six Dorsal Vertebræ, all of which were restored to their natural Situations in the Spinal Column, by the Process employed for that Purpose.* Communicated by EDWARD HARRISON, M.D. F.R.A.S. ED. &c.

COLONEL SIBTHORP, æt. 40, had the misfortune to be overturned in his carriage, on the 23d of February, 1821. The upper part of his back was much bruised, and he has, ever since the accident, been paralytic in all his limbs, especially in the left arm and leg. In both the members last mentioned, as well as in the trunk of his body, the sense of feeling is greatly impaired: it is chiefly defective on the right side. Upon examination, I made the following observations: viz.—I found that the third dorsal vertebra had been forced considerably inwards; the fourth nearly retained its natural position; the next five were likewise sunk so much below their proper line, that it was very difficult to feel even their spinous processes. By strong pressure upon the part, a hard resistance could be distinguished, but it was impossible to ascertain its form, or of what it consisted.

I caused a machine to be made of thin slips of fir, similar to those used for the support of fractured limbs, sufficiently large to cover the depressed vertebræ; this I had firmly attached to the back; directing, at the same time, that it should be worn constantly during the cure. The instrument in question was grooved deeply in a longitudinal direction, in such a manner as to produce an hollowness between it and the spinal column, which might thus leave the affected part wholly without external pressure. After all was properly arranged, I caused the spine to be stretched daily, for an hour at a time, in order to draw out, and in some degree to separate, as it were, the vertebræ from each other. This operation was performed by means of the shoulders being pulled by one person placed behind the head, and the feet at the same time by another, in opposite directions; the colonel all the while lying upon his back. During the period that this process was going on, I continued to make, with my own hands, firm pressure upon the sternal ends of the ribs, first on the one side, and then on the other. By this contrivance they were forced, at the other end, to act powerfully upon the depressed vertebræ. This was done with a view to drive them outwards, and towards their proper situations in the column.

*September 27th,* 1821.—The third vertebra is entirely replaced; the other five are sufficiently elevated for their spinous processes to be easily distinguished, and also the turn of them, which was made towards the right side, occasioned, as there is every reason to believe, by the fall above mentioned. The Colonel feels warmer, and thinks that he has greater firmness and activity in the muscles of his arms. The lower extremities have been little used, as he reclines continually upon a crib or couch.

*October 10th.*—A progressive advancement of the affected vertebræ hath been distinctly perceived, after every operation. In six weeks, they were declared to have entirely regained their natural stations, and the process was discontinued. In proportion as the bones advanced towards the surface, the feeling and the activity of the patient's limbs were increased. The treatment was finished November 20th. He then no longer made constant use of the crib, but occasionally amused himself by gently walking about, as well as in the indulgence of moderate exercise in a carriage.

To give some idea of the weak state in which Colonel Sibthorp found himself previously to his entering upon my plan of cure, and also of the very material alteration visible in him subsequent thereto, I need only mention that, upon his calling one day, before the treatment commenced, in company with his friend Mr. Mainwaring, upon Mrs. Harrison, at my house in Holles-street, so feeble did he feel himself, that he requested to be excused from making the exertion of accompanying Mr. Mainwaring up stairs into the drawing-room, being anxious, as he himself expressed it, to be spared the labour and fatigue of ascending even a single flight of stairs: this, of course, he refrained from attempting, and accordingly remained, during their visit, in the apartment below. Whereas, the treatment ended, he not only did not hesitate to walk on foot from his own lodging in Duke-street, Portland-place, to visit me in

Holles-street, but moreover, without any objection, and with great ease, ascended the stair-case into the drawing-room; from whence Mr. Mainwaring and myself accompanied him to Mr. Brooks's extensive museum in Blenheim-street. Here the Colonel walked about for upwards of an hour and a half, enjoying at his ease the various specimens with which the different apartments abound; nor did he experience, as far as I could learn, the smallest inconvenience therefrom, either at the time or afterwards, upon his arrival at home.

His health was at that period, as indeed it had been from the time of his becoming my patient, in a perfectly good state; and in such it continued during the remainder of his stay in London. A few aperient medicines only were given to him while he resided in town, no other being deemed necessary.

He bore walking exercise, and driving over the stones, so well, that he decided, in an unlucky moment, to return to his seat in the country, at the distance of at least 130 miles.

The weather, which had been generally warm and fair until a day or two before his departure, became now cold, rainy, and boisterous.

I passed several hours with the Colonel on the last evening that he spent in town; the Rev. Humphrey Sibthorp and Mr. Mainwaring were of the party. Our conversation embraced various topics, and the Colonel took his full share in the discussions: he was then, as he had ever been during my attendance upon him, in good bodily health. For the truth of this assertion, I appeal to the gentlemen whose names I have just mentioned, more especially to the letter of his intimate friend Mr. Mainwaring, who had been his constant companion while in London.

From the moment of our separation, of course, all control on my part over the patient must have been necessarily suspended, and consequently I cannot, in reason, stand implicated: neither, in justice, ought I to be charged with any malady that might have assailed him subsequently thereto. It will, therefore, be sufficient for my purpose to show that Colonel Sibthorp continued well during the time that he was under my treatment, and that, moreover, he left me in the enjoyment of good health.

Having proved these facts, and placed them in their proper light, it forms no part of my business to account for the fatal indisposition which commenced after his departure from town. This distressing event, notwithstanding the late exculpatory publication of a medical attendant,* is still involved in *thick* darkness.

My second professional visit to Colonel Sibthorp was made at the Waterloo Hotel, Regent-street, about a week before he

* See *Medical Repository* for November 1822.

became my regular patient. The moment his back was uncovered, I was so forcibly struck with the extraordinary cavity between his shoulders, that I requested that Mr. Mainwaring might be invited to see it. Upon that gentleman's entering the room, I pointed out to him, as well as to the Colonel's servant, Thomas Whaley, the unnatural sinking of the spine. I then proceeded to trace before them the six depressed dorsal vertebræ, enumerating their names, and explaining their true situations. At my desire, Mr. Mainwaring examined the spine very carefully. He made many inquiries of the Colonel and of me, with a view to satisfy himself of the true nature and extent of the injury which had been inflicted.

During the whole time that the Colonel was under my professional management, these investigations were frequently repeated, so that the extent of the changes and gradual elevation of the vertebræ often became the subject of conversation.

My nephew, Dr. Richard Harrison, accompanied me on one occasion to visit the Colonel; besides whom, Mr. Parker, one of his regimental surgeons, frequently assisted in the process. Both these gentlemen carefully examined the spinal column, and took part in the discussions; they are, therefore, fully qualified to state their opinions of the progressive improvement that took place in the arrangement of the depressed vertebræ, and to say how far the Colonel's health was affected by the treatment he underwent. With a view to obtain their sentiments, as well as those of Mr. Mainwaring, and of the Colonel's servant also, the circular letter, of which the following is a copy, was sent to each of them: viz.—1. To Mr. Mainwaring, and to his servant. 2. To Thomas Whaley. 3. To Mr. Parker. 4. To Dr. Richard Harrison.

(CIRCULAR.)

Dear Sir,—I have particular reasons for desiring to ascertain your opinion of Colonel Sibthorp's case, and of my treatment of it, during the time that he remained under my professional care in London, in as far as they came under your personal observation. You will, therefore, do me a favour by answering the subjoined questions: viz.—

1st. Can you recollect, at your first visit, what was the length, depth, and width, of the depression in the spinal column?

2d. Did you know at the time, and can you now remember, how many of the vertebræ were actually depressed?

3d. Can you remember whether they gradually rose under the operation, till they got to be of equal height with the other vertebræ; or whether they remained immoveably fixed, and quite stationary, during the whole period of my attendance?

4th. Did the Colonel's health appear to suffer from the process adopted, or the confinement; and what was the state of it when he left London?

You will greatly oblige me by adding any remarks that strike you as bearing upon the case or the treatment, although the questions may not, apparently, include them.

I am, dear Sir, &c. &c.

EDWARD HARRISON.

To the above letter, the following answers were returned by the parties to whom it was addressed respectively: viz.

"*Coleby; Oct. 23d*, 1822.

"Dear Sir,—In answer to your letter, which I received lately through the hands of Mr. Edward Chaplin, requesting me to state any observations I may have made on the treatment of my greatly lamented friend, Colonel Sibthorp, during the time he was under your care in London, I have to state that I can have no objection to mentioning the nature of my sentiments thereon, the results of an anxious, though unskilful, attention: at the same time observing, that, unless *through necessity*, it would be with great reluctance that I should see my name publicly brought forward in a controversy between two people, to both of whom I am under professional obligations. It was, I think, on the second visit of yourself to Colonel Sibthorp, (being from home the first,) that you pointed out to me the depression of the Colonel's spine. The vertebræ depressed appeared to be about six, with one rather higher in the centre; but I am unable, at this time, to state the exact length, width, or depth, of the depression: it was, however, evident to both me and his servant that, after having submitted for some time to the prescribed treatment, they gradually rose to a level with the rest of the back bone. In regard to the Colonel's general health, it was my decided opinion that, so far from suffering from the process adopted, or from the confinement, it appeared to improve, and his appetite to be much benefited; so much so, that I had the greatest hopes of seeing him perfectly restored. I must however, in fairness, state, that, though the Colonel gave us credit for reports about his back, he often declared to me that he could perceive no improvement in the use of his left arm or hand, or in the numbness of his right side, which he said remained the same.

"With regard to the Colonel's melancholy illness, subsequent to his return, I cannot, in my mind, in any degree, attribute it to the process or confinement he underwent in London; but, on the contrary, to strong exercise on his weak and debilitated constitution, as he had a similar attack on his journey to London, which commenced on the second day, at Biggleswade, with a violent head-ache, attended with sickness that evening, which prevented him taking advice about his back for a full week. The most serious attack that I remember his having, of that kind, was after you had set him at liberty, on his imprudently dining at the Piazza Coffee-house, and going to the play afterwards;* which was attended, as before, with what he often complained of—a constipation of the bowels. Exercise seemed, generally, to be injurious to him; and, though he bore well the two or three

* The transgression alluded to was committed without my knowledge, and the effects arising therefrom must have been very transient to have escaped my observation.

drives he took in the Regent's Park with your permission, yet longer journeys, and even short distances in a hired carriage over the London pavement, he appeared to suffer from. In my opinion, he died from the effect of the accident on an already shattered constitution, which no medical skill could prevent, and which, by his own account to me, seemed to paralize his intestines.

"I have now, I trust, answered your several questions with perspicuity and candour; and, with best compliments to Mrs. Harrison, I remain, dear Sir, your's truly,

"CHARLES MAINWARING."

"*Lincoln; November 24th,* 1822.

"Honoured Sir,—Having received some instructions* from you, through the hands of Mr. Mainwaring, relating to the case of my late master, Colonel Sibthorp, I take this opportunity of answering them, as well as my abilities will allow me.

"In regard to the first three questions, it is not in my power to give any answer to;† but, in regard to his health during the time he was under your care, I can, with confidence, say I had not known it better for a long time before; and, further, to say I recollect my late master having a very severe attack of the head-ache and sickness during the time he was at Warren's Hotel, before you begun with him, the same as he had when he got home.

"Honoured Sir,

"I remain your much obliged and humble servant,

"THOMAS WHALEY."

"34, *Devonshire-street, Portland-place; Nov. 9th,* 1822.

"Dear Sir,—I have to acknowledge the receipt of your circular letter of the 6th instant, and to assure you it will give me great pleasure if, in replying to your questions respecting the case of my much-lamented friend Colonel Sibthorp, I can afford you any useful information.

"At my first visit to Colonel Sibthorp, when I examined the spine, there appeared a very considerable depression of about four or five‡ of the superior dorsal vertebræ; and, to the best of my recollection, the uppermost ones were more depressed than the lower. This was in the month of October, 1821.

"I often witnessed, and several times assisted in, the operation which you deemed proper for the restoration of the parts; and I am of opinion

* A copy of the circular letter was delivered by Mr. Mainwaring to Thomas Whaley.

† The silence of this correspondent in regard to the fissure and depression of the spine, is not a little extraordinary, after what he had daily witnessed, and repeatedly asserted, in conversation, to persons of respectability and veracity, both in London and also after his return into Lincolnshire.

‡ The apparent contradiction between the case and this letter is satisfactorily explained by comparing the dates. Mr. Parker's first visit to Colonel Sibthorp took place, I believe, later than the 10th of October, at which time the restoration of one of the six depressed vertebræ is particularly reported to have taken place.

that the adjustment of the vertebræ which took place was to be wholly attributed to the plan adopted.

"I examined the Colonel's spine frequently during the process, and witnessed its gradual recovery, and I can most confidently assert that the depression, which was so evident at my first visit, was not perceptible when he left London for Lincolnshire.

"His general health, so far from suffering, evidently improved under the treatment. His appetite was very good, and the digestive organs seemed to perform their office perfectly. He increased in bulk, and his looks indicated that return of vigour which he repeatedly mentioned to me he experienced.

"I shall have great pleasure in giving you any further information that I may recollect, should you need it; and I am, my dear Sir, yours most truly, THOMAS PARKER, *Surgeon.*"

"*To Dr. Harrison, Holles-street, Cavendish-square.*"

"*Argyle-street; Dec. 14th,* 1822.

"My dear Uncle,—I wish I were able to give a more satisfactory answer to your questions respecting the late Colonel Sibthorp, than I am capable of doing, from having only seen him once. There was at that time a very considerable depression of the superior dorsal vertebræ, but, with respect to the exact number depressed, I am unable, at this distant period of time, to charge my memory, notwithstanding I examined the spine with great attention; but the depression was so very strongly marked, that it must have been obvious even to the eye of the most inexperienced observer.

"The Colonel did not complain, nor appear to suffer any inconvenience from the mode of practice you pursued. But I must confess I left him fully impressed with the idea that you could not succeed in removing the depression. This opinion I frequently expressed to Dr. Hutchinson, to whom I gave a description of the case, and mode of treatment you had adopted.

"Dr. Hutchinson, who had frequently accompanied you in your visits to other patients,* and was fully convinced of the efficacy of your practice, still coincided with me that, in Colonel Sibthorp's particular case, you would fail. It was with very great surprise I learned that the Colonel's spine had resumed its natural form.

"I remain, my dear uncle, yours very sincerely,

"R. HARRISON."

"*Dr. Harrison, 7, Holles-street.*"

---

* I expected, when I last addressed the profession, to have unfolded my particular opinions before this time, in some of the medical journals. Though various circumstances impeded the execution of my wishes, I never once refused, when called upon, to explain my sentiments to the faculty, and have invited different members of it to witness, at the bed-side, the mode of treatment pursued. More than twenty medical men have, at different times, accepted the offer. Out of this number, not fewer than seven, who received all their instructions from me, have already embarked in the new practice, with my approbation, in as many counties of England, Wales, and Scotland.

"Dr. Hutchinson, the gentleman referred to, was particularly recommended

It appears, from the preceding history of this case, which was drawn up and inserted in my common-place book, on the evening of September 27th, 1822, as well as from the returns made to my circular letter, that, when Colonel Sibthorp first applied to me, he laboured under the effect of great depression in six of the dorsal vertebræ.

This disordered state of the spinal column is supported by the concurring testimony of so many competent and disinterested witnesses, that the fact is fully established. They also maintain, unanimously, that this depression was entirely removed by the process adopted for that purpose.

This last opinion (as has already appeared by Mr. Joseph Swan's own statement,*) was further confirmed, on dissection, by the medical gentleman who attended the Colonel in his last illness, assisted by other neighbouring practitioners.

Fortunately for my professional character, in this dissection, no sort of mischief, arising from my mode of treatment, could be detected; and the new practice maintains its reputation, after the most rigorous scrutiny. So far from it, this investigation produced a confirmation of the innocence, and of the efficacy, of the method which had been adopted on this occasion. It proved that no sensible hurt had been inflicted upon the spinal cord,† either by the long-continued compression, or by the treatment subsequently employed for its removal.

The inference to be drawn from these premises is truly important, and, as it rests upon unquestionable authority, is entitled to every degree of credit.

I was desirous to place these facts upon a firm basis, on account of the use to which they will be hereafter applied; and because Mr. J. Swan has had the boldness to give them an unqualified denial, on his own individual responsibility. "At this time, May 1821," he observes,‡ "I again examined the spine, but could not perceive any irregularity in the bones; and, indeed, I never could, though I had examined it frequently with the greatest care. When the accident first happened, I could never perceive any crepitus, or other symptom that could induce me to suppose that *the vertebræ were fractured*, unless the pain on pressure of his left side would lead to such a supposition." Again, "For myself," continues this gentleman,§ "I have no hesitation in thus publicly avowing that I all along expressed to those that asked me, that, in my opinion, it was utterly impossible thus to have restored the parts to their naby me to undertake, upon his own account, the treatment of spinal diseases in London. Had this learned and excellent physician remained in England, he would, I have reason to believe, have followed my advice.

* See SWAN *on the Nervous System*, p. 79.

† *Ib.* ‡ *Ib.* p. 65. § *Ib.* p. 70.

tural position, supposing any dislocation had really taken place, *which, however, I was confident was not the fact.*"

We are told that Dr. Cookson and Mr. Henry Swan were in the habit of seeing Colonel Sibthorp, as well as Mr. J. Swan: how, then, has it happened that the testimony of neither of these gentlemen is brought forward in support of the above statement? However satisfactory and convincing Mr. J. Swan's declaration may be to himself, that there existed "no irregularity in the bones," I am convinced that the public would have placed greater confidence in his assertion had it been corroborated by (as we must naturally think that it should have been,) the concurring testimony of his two coadjutors, above mentioned.

It appears to me, from many circumstances, that these gentlemen were misled by a chimerical hypothesis of their own creation to neglect the proper inquiries, while they could have been made useful to their suffering patient. The same blind confidence led them, as the dissection has shown, to misconceive the true nature of the disorder,* and to adopt a method of treatment, the total inefficacy of which was clearly demonstrated by the fatal termination of the case. Under the mistaken idea that a blood-vessel, ruptured by the violence of the accident, had poured its contents into the spinal canal,† in quantity sufficient to oppress the inclosed cord and interrupt its functions, they were unfortunately induced to lower, by bleeding, salivating with mercury, and other debilitating means, a constitution already too much exhausted. This is no unreasonable conclusion, since the Colonel had, on so many former occasions, succeeded in removing his head-ache and the accompanying symptoms by rest alone, or by mild remedies properly administered.

Had these gentlemen confined their practice, in this instance, to plain and rational indications, very different might, I conceive, have been the result!

I have, I think, made it sufficiently appear, from the preceding narrative, as well as from the various letters which I have herewith produced, that Colonel Sibthorp left London, upon his return into Lincolnshire, December 3d, in good bodily health. Under these circumstances, therefore, I feel myself exonerated, as I have already declared, not only from all blame, but likewise from all responsibility, as to every thing that took place subsequently to his departure from London.

"He reached home," adds Mr. J. S.‡ "on the 5th December, and I visited him on the 7th. He, at that time, complained of vomiting, head-ache, impaired vision, and constipation of the bowels. He said his left arm was nearly in the same state as

* Swan *on the Nervous System*, p. 66. † *Ib.* ‡ *Ib.* p. 70.

when he went to London; and, if it was improved at all, it was not in a greater degree than he thought it would have been in the same time, provided nothing had been done. He seemed desirous of trusting himself to the *vis medicatrix naturæ, as, in consequence of what had recently occurred, he had, with very good reason, a great aversion to the interference of art.*"

Such are the terms of the conversation mentioned by Mr. J. Swan as having passed between him and Colonel Sibthorp. Let us now see how far they agree with other statements, and are supported by collateral proofs.

On the 10th of the following month (viz. January), when suffering from what he called a bilious attack, and before any of the Lincoln faculty had been consulted, the Colonel drew up his own case, for my professional opinion. I am convinced, from this, as well as from other circumstances, that he was well satisfied with the treatment he underwent in London, and that he would not, after his return into the country, have so long confided his disorder to the "vis medicatrix naturæ," had he placed much confidence in the medical skill of the professional advisers who afterwards attended him. At the same time that he wrote to me, he expressed himself in another letter, addressed to Mrs. Harrison, in the terms following: viz.—

"I owe you many thanks for your obliging inquiries after me, and which I have some days meditated to reply to, but have been variously prevented. You will, I know, have pleasure in hearing that *my back continues all right, and my arms gain strength, and increase in size.* I have no fear of any relapse as to the back, though I still think it as well to be careful, and therefore continue to wear the shield and belt. Want of exercise, and this very extraordinary season, have made me feel rather more than usually bilious; and, as you were so kind as to speak of this in your letter, I have inclosed a statement of my feelings for Dr. Harrison, which I will trouble you to give to him.

"Believe me, my dear Madam, your's, &c.

"CONINGSBY SIBTHORP."

"*Canwick; 10th January,* 1822."

The following is a copy of the statement alluded to in the foregoing letter to Mrs. Harrison: viz.—

"My dear Sir,—I am encouraged, by a few lines of kind inquiry from Mrs. Harrison, to request your assistance in fighting something of a bilious attack, which of late has been very troublesome. It generally appears in the shape of a violent head-ache, coming on towards evening, and increasing in the morning, when I feel a nausea and sickness at the stomach, a strong inclination to retching, but seldom bring any thing from the stomach, until I take either tea or warm water; and then only what I have just at the time swallowed. The sensation of sickness and the feeling of a wish to relieve myself still remain, until I am able to vomit so effectually as to get rid of the bile. Frequently, on former similar occasions, after being sick, in the course of an hour or two my head-ache would disappear: it now often remains twenty-four and forty-eight hours, and even longer. I feel no pain in the stomach, but sometimes a sensation of fulness; and my bowels are costive, so much so now, as seldom to have a free motion without the assistance of medicine.

"I could once eat and drink any thing with impunity, but am now obliged to be most careful. The pain in my head is not confined to one part, but is sometimes just over the eyes, sometimes at the back of the neck, and accompanied with throbbing, and lately a great dimness of the eyes.

"I beg you to excuse my being thus circumstantial and prosing, but I should be truly glad to be relieved of this disagreeable malady. I will not, however, occupy more of your valuable time than to say I am your grateful and obliged friend,

"CONINGSBY SIBTHORP."

"*To Dr. Harrison,*

*7, Holles-street, Cavendish-square.*"

Whoever is at the trouble to compare, dispassionately, the account given by Colonel Sibthorp of his own feelings, and contrasts it with that of Mr. J. Swan, cannot fail, I think, to be struck with their disagreement. He will, in that case, be inevitably led to conclude, either that the Colonel told one story to his surgeon and another to Mrs. Harrison and myself, or that the conversation which Mr. J. Swan states to have taken place had been, unintentionally perhaps, misrepresented and perverted, through the strong prepossessions of the narrator.

To the friends of this excellent man, whose memory will be long cherished among them, it is superfluous to say a word in defence of his undeviating veracity. To all others, I boldly maintain that his candid and honourable mind was incapable of dissimulation, much more of uttering a palpable untruth, to serve any purpose whatever.

Having, I trust, satisfactorily proved that a considerable depression of the upper dorsal vertebræ was actually produced by his overturn, the symptoms under which Colonel Sibthorp suffered, immediately after the accident, will admit of an easy and satisfactory explanation.

Six dorsal vertebræ were forcibly driven inwards by the fall, so as to press upon the spinal marrow in the canal within them. This important organ was, in consequence of the concussion, impeded and interrupted in its functions. One of the most important of these (viz. muscular motion,) became alarmingly impaired, so long as the pressure remained. After its removal, the nervous energy being conveyed more freely to the limbs, they soon acquired greater firmness, activity, and vigour. The amendment in the arm is particularly referred to in the Colonel's own letter, and displayed itself more and more every day while he remained in London.*

Deeply as I lament the untimely end of Colonel Sibthorp, and satisfied, as I am, that it took place from causes unconnected with my manner of treatment, the case is highly interesting, and leads to important conclusions. We are instructed by the dissection that, at the time of death, the vertebræ, the canal, and spinal cord, were all of them in their natural situations. The pathological inferences to be deduced from this case are entitled to the greatest attention, because the foregoing statement has proved that the vertebræ will admit of being driven out of their places, and again restored, without occasioning either lasting mischief to the spinal cord, or even any visible alteration of its structure.

In all former injuries of the spine from external violence, the unfortunate sufferer has been abandoned to his hard fate, and no effort been hazarded for his relief. Encouraged by the successful issue of this case, I should recommend that, on similar occasions, a trial be made, as speedily as possible, to replace the luxated vertebræ, and thereby free the spinal marrow from the consequences of injurious pressure. When the attempt proves successful, a cure may reasonably be anticipated: provided, on the other hand, that it should fail, no inconvenience will follow the experiment, as the disorder, left to itself, has always terminated fatally.

The conclusions to be drawn from the preceding narrative are—

1st. That, when Colonel Sibthorp became my patient, September 27th, 1821, six of the upper dorsal vertebræ were much depressed, and sunk below their proper situation in the spinal column.

2dly. That all the affected vertebræ were admitted, November 20th, to have been wholly restored and raised to their natural level.

3dly. That during the cure, and at the time of departure for Lincolnshire, the Colonel was in excellent health.

4thly. That he, moreover, gained flesh, strength, and activity, during the treatment adopted by me.

5thly. That it was proved, by the post-mortem examination, that the vertebræ which had been displaced, the spinal marrow within them, and also the connecting ligaments, were, at the time of death, in a sound and healthy condition.

---

* The power that I possess of altering the arrangement of the distorted vertebræ and ribs will be more fully explained in my Essay on Subluxations of the Spine. This work is already in considerable forwardness, and will be illustrated with descriptive engravings.

